# Role of *Abca7* in Mouse Behaviours Relevant to Neurodegenerative Diseases

**DOI:** 10.1371/journal.pone.0045959

**Published:** 2012-09-24

**Authors:** Warren Logge, David Cheng, Rose Chesworth, Surabhi Bhatia, Brett Garner, Woojin Scott Kim, Tim Karl

**Affiliations:** 1 Neuroscience Research Australia, Randwick, Australia; 2 Schizophrenia Research Institute, Darlinghurst, Australia; 3 School of Medical Sciences, University of New South Wales, New South Wales, Australia; 4 Illawarra Health and Medical Research Institute, University of Wollongong, New South Wales, Australia; 5 School of Biological Sciences, University of Wollongong, New South Wales, Australia; 6 School of Psychology, University of New South Wales, New South Wales, Australia; University of South Florida, United States of America

## Abstract

ATP-binding cassette transporters of the subfamily A (ABCA) are responsible for the translocation of lipids including cholesterol, which is crucial for neurological function. Recent studies suggest that the ABC transporter ABCA7 may play a role in the development of brain disorders such as schizophrenia and Alzheimer’s disease. However, Abca7’s role in cognition and other behaviours has not been investigated. Therefore, we characterised homozygous *Abca7* knockout mice in a battery of tests for baseline behaviours (i.e. physical exam, baseline locomotion and anxiety) and behaviours relevant to schizophrenia (i.e. prepulse inhibition and locomotor response to psychotropic drugs) and Alzheimer’s disease (i.e. cognitive domains). Knockout mice had normal motor functions and sensory abilities and performed the same as wild type-like animals in anxiety tasks. Short-term spatial memory and fear-associated learning was also intact in *Abca7* knockout mice. However, male knockout mice exhibited significantly impaired novel object recognition memory. Task acquisition was unaffected in the cheeseboard task. Female mice exhibited impaired spatial reference memory. This phenomenon was more pronounced in female *Abca7* null mice. Acoustic startle response, sensorimotor gating and baseline locomotion was unaltered in *Abca7* knockout mice. Female knockouts showed a moderately increased motor response to MK-801 than control mice. In conclusion, *Abca7* appears to play only a minor role in behavioural domains with a subtle sex-specific impact on particular cognitive domains.

## Introduction

ATP-binding cassette (ABC) transporters are classified into seven subfamilies according to their structure and sequence homology. The subfamily A (ABCA) is responsible for the translocation of a variety of lipids including cholesterol across membranes. There is also evidence to suggest that they might contribute to regulation of neurogenesis, phagocytosis and host defense [1]–[3]. Although the brain is extremely enriched in lipids little is known about the role that ABCA transporters play in cognitive function. Emerging evidence indicates that tight regulation of brain cholesterol homeostasis is crucial for neurological function and that abnormal cholesterol homeostasis can contribute to neurodegeneration [4]. Thus, modulation of endogenous ABCA transporters could potentially have a significant impact on brain function and susceptibility to neurodegenerative diseases.

The ABCA transporter ABCA7 is expressed in the mouse brain with particularly high expression in hippocampal and cortical neurons as well as microglia [4]–[6]. A potential role of *ABCA7* in the development of brain disorders has recently been suggested. A single nucleotide polymorphism (SNP) of *ABCA7* has been found to be associated with schizophrenia [7]. Furthermore, a deregulation of apoptosis in cortical regions of schizophrenia patients has been reported [8]. Apoptosis is a key process in shaping neuronal circuits and functions. In this context, ABCA7’s role in phagocytosis [9] might be important to maintain tissue homeostasis and prevent the release of potentially cytotoxic or antigenic molecules from dying cells during apoptosis. In Alzheimer’s disease (AD), common variants in *ABCA7* have been linked to AD and a recent meta-analysis provided compelling evidence that the SNP rs3764650 represents a new AD susceptibility locus [10]. Furthermore, modulating pathways that regulate cholesterol homeostasis seem to affect amyloid precursor protein (APP) processing and the production of β-amyloid peptides, which are neurotoxic and pro-inflammatory, impair memory and represent a major constituent of cerebral amyloid plaques associated with AD [4], [11].

Kim et al. developed a viable homozygous *Abca7* knockout mouse (i.e. *Abca7* ^−/−^) with no remarkable phenotype except a reduction of white adipose tissue mass in knockout mice and lower total serum and high-density lipoprotein cholesterol levels in female *Abca7* ^−/−^ mice [5]. Importantly, the behavioural phenotype of this mouse model has not been determined to date. Therefore, we employed a battery of tests for baseline behaviours (i.e. physical exam, locomotion in the open field and anxiety in the elevated plus maze) as suggested by Crawley and others [12], [13] and also considered behaviours relevant to schizophrenia (i.e. prepulse inhibition and locomotor response to psychotropic drugs in the open field; [14]) and Alzheimer’s disease (i.e. cognitive domains using the novel object recognition task, the Y maze, the fear conditioning paradigm, and the cheeseboard; [15]) in *Abca7* ^−/−^ mice to explore whether *Abca7* influences a broad range of behavioural domains. This strategy will provide information about the potential involvement of *Abca7* in a variety of behavioural domains with a particular focus on those relevant to neurodegenerative diseases.

## Results

Male *Abca7^−/−^* mice exhibited wild type-like sensory abilities and neurological reflexes and showed no differences in motor abilities or motor function in the accelerod (i.e. latency to fall of the rotating rod), the pole test (i.e. latency to climb down the pole to reach platform), the wire hang test (i.e. latency to fall) and the beam walking test (i.e. latency to cross squared beams) (all *p*–values >.05; Table 1). The body weight was similar across genotypes (*p*>.05; Table 1).

**Table 1:** Motor function/coordination in the physical exam and body weight.

	Male		Female	
	WT	*Abca7^−/−^*	WT	*Abca7^−/−^*
Accelerod: Latency to fall of rod [s]	149.8±15.5	146.5±14.0	166.9±9.5	155.1±14.6
Pole test: Latency to reach platform [s]	26.8±4.5	22.6±3.9	34.6±9.7	32.6±4.1
Wire hang test: Latency to fall [s]	18.5±2.0	20.3±3.6	33.1±4.9	38.3±4.8
Beam walking test: Latency to cross squared10 mm^2^ beam [s]	15.5±1.2	16.0±1.6	18.2±4.1	15.4±2.2
Beam walking test: Latency to cross squared5 mm^2^ beam [s]	13.1±1.6	12.8±1.3	13.4±1.9	12.3±1.2
Body weight [g]	31.8±0.5	31.5±0.8	28.2±1.1	26.8±0.8

### Anxiety

In the EPM, locomotive activity (i.e. closed arm distance) and the occurrence of anxiety-related behaviours (i.e. time in open arm, open arm entry ratio, and frequency of *stretch attend postures*) was similar in WT and *Abca7* ^−/−^ mice (*p*–values >.05 for all parameters investigated), indicating that *Abca7* deficiency had no impact on EPM locomotion or anxiety (Table 2). Female mice showed a somewhat lower level of risk assessment (i.e. frequency of *stretch attend postures*) than male mice [two-way ANOVA for ‘sex’: F(1,38) = 9.6, *p* = .004; Table 2].

**Table 2:** Locomotion and anxiety-related behaviours in the elevated plus maze.

	Male		Female	
	WT	*Abca7^−/−^*	WT	*Abca7^−/−^*
Closed arm distance [cm]	911.3±37.6	849.7±48.3	968.9±40.8	881.5±74.6
*Stretch attend postures* [n]	11.0±1.2	13.5±1.9	8.1±0.7^##^	8.4±1.5
Open time [s]	19.6±3.2	18.6±1.9	15.2±1.7	15.3±2.5
Open entry ratio [%]	26.0±3.1	25.3±1.7	24.2±2.2	25.1±3.8

Distance travelled in enclosed arms, frequency of *stretch attend postures*, time spent on open arms (open time) and number of open arm entries as a percentage of total arm entries (open entry ratio) are shown. Significant one-way ANOVA split by ‘genotype’ effects of females *versus* males of the corresponding genotype are indicated by ‘#’ (^##^ *p*<.01).

### Cognition

#### Y-maze

**Figure 1:**
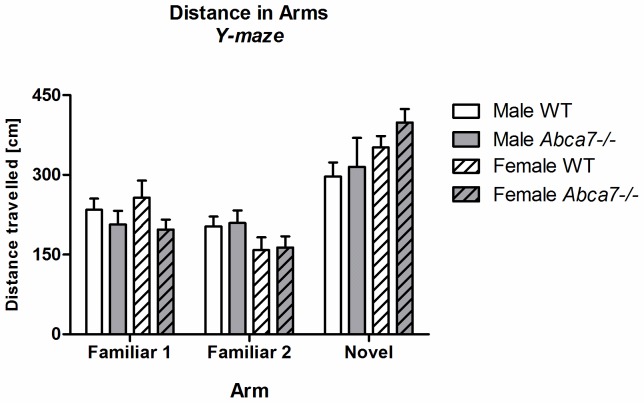
Recognition of familiarity in the Y-maze. Overall arm distance [cm], in familiar (familiar 1 and familiar 2) and novel arms are shown.

Test animals of both genotypes distinguished between the three arms (‘arm type’: familiar arm 1, familiar arm 2, and novel arm) in the test trial [F(2,80) = 26.7, *p*<.0001; no interaction] and developed a preference for the novel arm over the familiar arms [simple contrasts for arm distance: novel arm *versus* familiar arm 1: F(1,40) = 24.0, *p*<.001– novel arm *versus* familiar arm 2: F(1,40) = 49.1, *p*<.001; Fig. 1]. Short-term memory was not affected by *Abca7* deficiency as novel time [F(1,40) = 3.0, *p*>.05] and novel distance [F(1,40) = 0.9, *p*>.05] were not different between genotypes (Table 3).

**Table 3:** Cognitive performance in the Y-maze (YM) and novel object recognition task (NORT).

	Male		Female	
YM	WT	*Abca7^−/−^*	WT	*Abca7^−/−^*
Novel time [%]	34.7±2.5	40.8±5.5	40.7±2.3	47.9±4.7
Novel distance [%]	40.3±2.5	40.7±4.7	46.6±2.9	52.8±2.7
NORT				
Exploration of familiar object [s]	14.9±2.6	20.9±3.8	11.8±2.3	16.2±3.0
Exploration of novelobject [s]	20.6±2.7	16.9±2.0	21.8±4.7	19.1±2.8

YM: Time spent in the novel arm (novel time) and distance travelled in the novel arm (novel distance) as a percentage of the total measure [%] are shown as measures for short-term memory. NORT: The duration [s] of exploring (time spent *nosing* + *rearing*) the familiar and the novel object are shown as a measure of working memory.

#### Novel object recognition task

**Figure 2:**
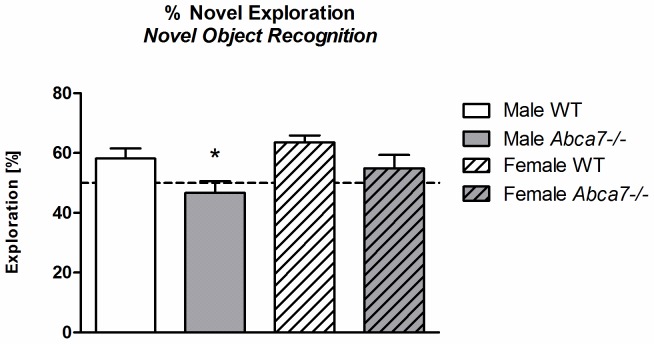
Novel object recognition. Percentage exploration [%] (i.e. duration of *nosing* and *rearing*) of the novel object (% novel exploration) is shown for males and females of both genotypes. Exploration of the novel object according to chance ( = 50%) is marked with a dotted line. Significant one-way ANOVA effects of *Abca7* ^−/−^ *versus* WT of the corresponding sex are indicated by ‘*’ (*p<.05).

Animals distinguished between the novel and the familiar object presented during the test trial as indicated by a significant effect of ‘object’ (i.e. novel *versus* familiar) on exploration time [F(1,33) = 4.8, *p* = .04; Table 3]. Importantly, the ability to recognise the novel object was genotype-dependent [interaction of ‘object’ with ‘genotype’: F(1,33) = 6.5, *p* = .02] as only WT mice showed a clear preference for the novel object [WT: F(1,17) = 14.9; *p*<.001 - *Abca7^−/−^*: F(1,16) = 0.1; *p* = not significant]. Impaired short-term novel object recognition memory of *Abca7* null mice was confirmed when analysing the % time mice spent exploring the novel object. *Abca7* ^−/−^ mice exhibited a reduced level of % novel exploration [F(1,33) = 7.8, *p* = .009] with males failing to develop a significant preference for the novel object (Fig. 2).

#### Fear conditioning

**Figure 3:**
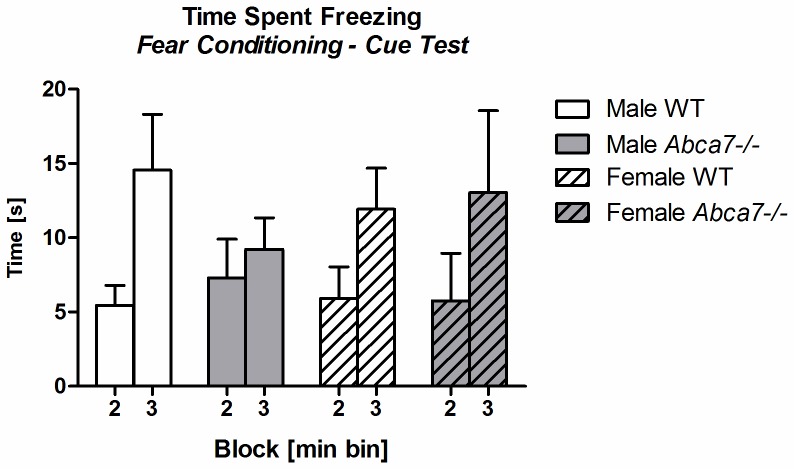
Fear conditioning in cue test. Time spent *freezing* [s] for 1 min before (block min: 2) and 1 min post CS onset (block min: 3) during the cue test is shown.

All animals responded to the electric foot shocks delivered during the conditioning phase (i.e. vocalization of all mice). The level of baseline *freezing* during conditioning (i.e. in the first 2 min before US presentation) was similar in all mice regardless of genotype and sex (all *p*–values >.05; Table 4). As expected, *freezing* during the first 2 min of the context test was significantly increased compared to the first 2 min of *freezing* in the conditioning phase regardless of genotype and sex [‘2 min’: F(1,36) = 39.5, *p<*.001; no interactions; Table 4]. The ability of *Abca7* knockout mice to associate the context with the US exposure during conditioning was WT-like, as the total time spent *freezing* during the context test was not significantly different across test groups (all *p*–values >.05; Table 4). In the cue test, test animals learned the association between US and CS. Mice responded to the CS presentation with increased levels of *freezing* after cue onset as confirmed by a significant main effect of ‘1 min block’ for time spent *freezing* one minute before and one minute post cue presentation [F(1,36) = 29.8, *p<*0.001; Fig. 3], indicating associative learning of WT and *Abca7^−/−^* mice.

**Table 4:** *Freezing* during conditioning phase and context test.

	Male		Female	
*Freezing* [s]	WT	*Abca7^−/−^*	WT	*Abca7^−/−^*
Conditioning phase (first 2 min)	4.4±2.3	1.9±0.8	0.3±0.2	0.7±0.4
Context test (first 2 min)	29.0±7.6	20.2±4.5	22.5±6.3	21.4±7.3
Context test (total 7 min)	86.3±19.2	86.6±13.4	93.3±18.2	94.6±17.9

Time spent *freezing* [s] is shown.

#### Cheeseboard

**Figure 4:**
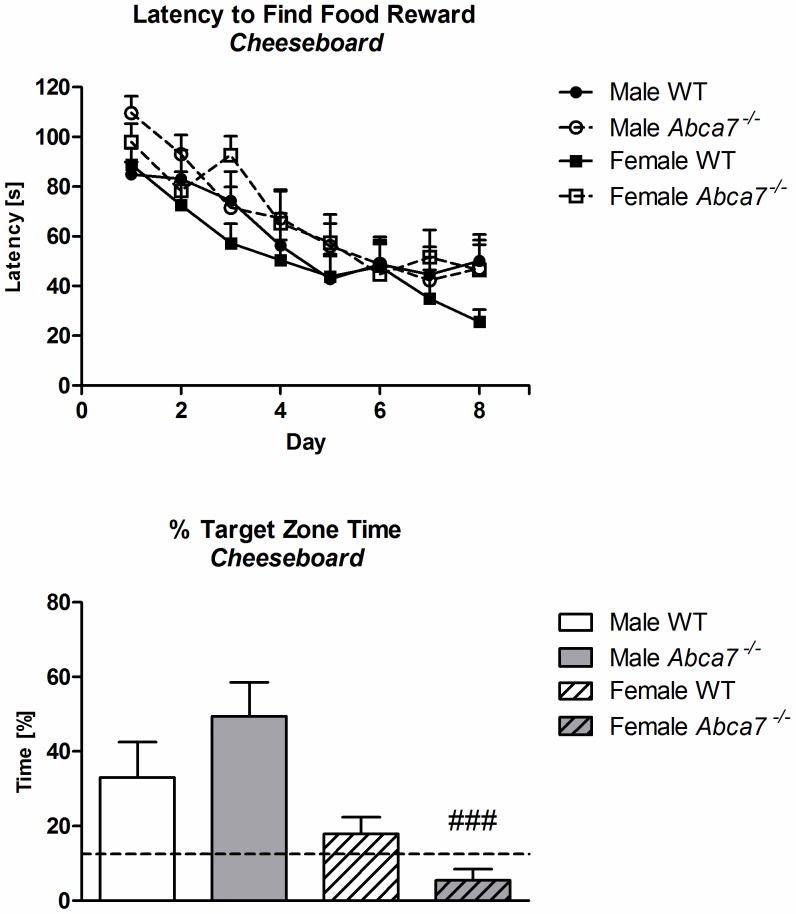
A+B Cheeseboard: A) Mean latency (averaged across three trials per day) to find the food reward [s] and B) percentage time [%] spent in the target zone during the probe trial (% target zone time). Time spent in the zone according to chance ( = 12.5%) is marked with a dotted line. There was a strong trend for an interaction of ‘sex’ with ‘genotype’ interaction in the probe trial (*p* = .05). Significant one-way ANOVA effects of females *versus* males of the corresponding genotype are indicated by ‘#’ (^###^ *p*<.001).

Mice of both sexes and both genotypes improved their performance to find the food reward over the first eight days of training, as evidenced by a significant effect of ‘day’ [F(7,273) = 25.3, *p*<.001]. There was no main effect of ‘genotype’ or ‘sex’ and no interaction on the latency to find the food reward, demonstrating that all mice equally learned the location of the reward (Fig. 4A). In the probe trial test, mice showed a preference for the target zone [t-test for target zone versus other zones: *p* = .001]. However, only male mice spent a significantly greater time in the target zone than chance (i.e. 12.5%) indicating that they had memorised the location of the food reward [two-way ANOVA for ‘sex’: F(1,39) = 17.0, *p*<.001]. Furthermore, a strong trend for an interaction of ‘sex’ with ‘genotype’ [F(1,39) = 4.0, *p* = .05] indicated that the impaired spatial reference memory of female mice in the probe trial was more pronounced in *Abca7* ^−/−^ females than WT females (Fig. 4B).

### Schizophrenia-relevant Behaviours

#### Startle response and prepulse inhibition

**Figure 5:**
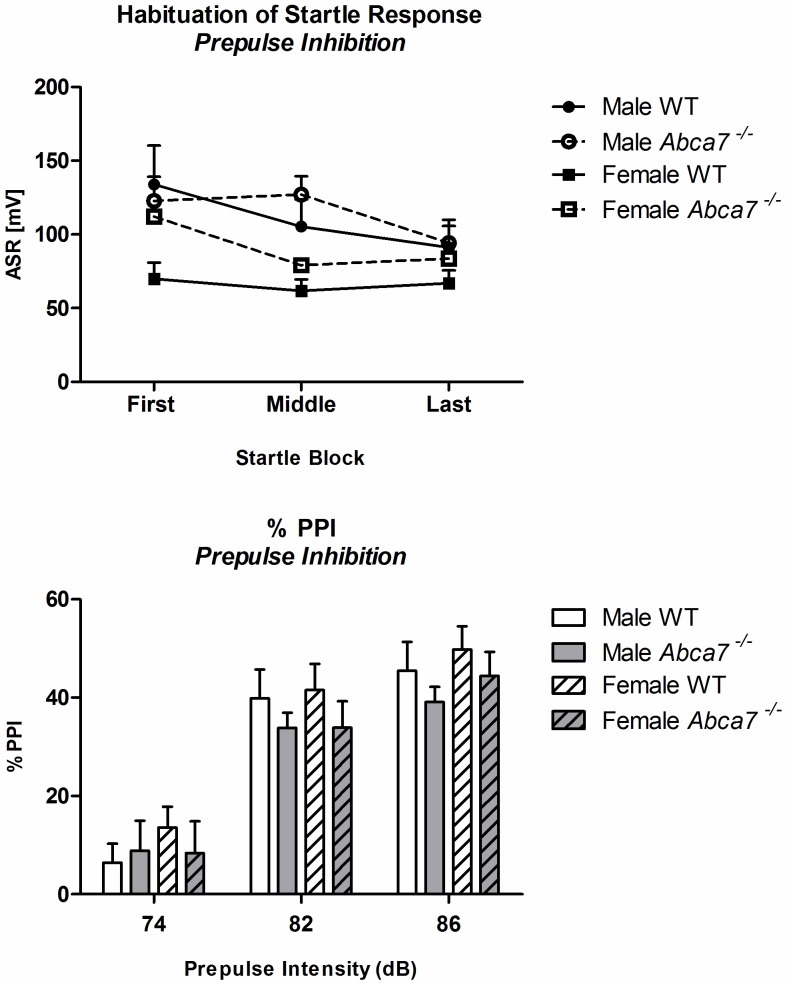
A+B: Habituation of startle response and sensorimotor gating: A) Habituation of the acoustic startle response to a tone stimulus of 120dB [millivolts] and B) % PPI for different prepulse intensities [%].

There were no effects of *Abca7* on the startle response of mice (*p*>.05) (Table 5). Female mice exhibited lower acoustic startle responses to a 120dB tone than male mice [F(1,39) = 7.0, *p* = .01]. Startle habituation over trials occurred in all mice [‘ASR block’: F(2,78) = 8.1, *p* = .001] and was similar across genotypes and sex (Fig. 5A). Prepulse intensities had a significant effect on % PPI [‘prepulse’: F(2,78) = 137.7, *p*<.001] with increasing prepulses causing more pronounced prepulse inhibition. Sensorimotor gating (i.e. % PPI) was not affected by *Abca7* or the sex of the test animals (all *p*–values >.05; Fig. 5B).

**Table 5:** Acoustic startle response to a 120 dB startle stimulus during PPI testing.

	Male		Female	
PPI	WT	*Abca7^−/−^*	WT	*Abca7^−/−^*
Acoustic startle response	110.2±18.3	114.7±11.4	69.5±8.4	88.6±10.9

Acoustic startle response [millivolts] is shown.

#### Baseline and MK-801 induced locomotion in the open field

**Figure 6:**
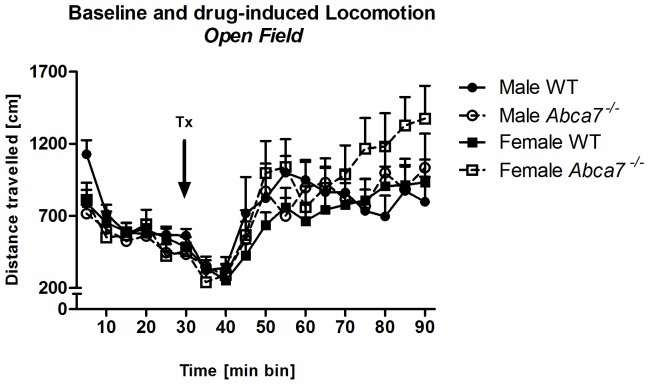
Baseline and MK-801 induced locomotion in the open field. Overall distance travelled [cm] (in 5 min blocks) in the open field in a 90 min session where animals received an i.p. injection of MK-801 (0.25 mg/kg body weight) after 30 min of baseline testing (indicated by black arrow). There was an interaction of ‘5 min block’ with ‘genotype’ in females (*p*<.05).

Mice across genotypes and sex showed similar locomotion rates (i.e. distance travelled) in the first 30 min of the OF test (all *p*–values >.05). Furthermore, all test animals showed habituation of their locomotive response to the open field in the first drug-free 30 min of testing [‘5 min block’: F(5,190) = 35.0, *p*<.001; Fig. 6], although habituation was influenced by ‘genotype’ and ‘sex’ [interaction of ‘5 min block’ with ‘genotype’ and ‘sex’: F(5,190) = 3.1, *p*<.01]. However, this was first of all due to increased baseline locomotion of WT males in the first 5 min block compared to *Abca7^−/−^* males and all females (Fig. 6).

As expected, MK-801 stimulated locomotion of all test animals [‘5 min block’ for 90 min OF test session: F(17,646) = 17.5, *p*<.001; Fig. 6]. An interaction of ‘5 min block’ with ‘genotype’ [F(17,646) = 1.9, *p* = .02] was based on increased locomotion levels of female *Abca7* knockout mice in the later test stages [‘5 min block’ by ‘genotype’ interaction: females: F(17,340) = 17.4, *p*<.05– males: not significant].

## Discussion

*Abca7* null mice have normal sensory abilities, neurological reflexes and motor functions and show wild type-like anxiety behaviour and sensorimotor gating. Short-term memory, fear-associated conditioning and spatial learning were also unaltered in *Abca7^−/−^* mice. However, deficiency in *Abca7* disrupted novel object recognition in male mice. Furthermore, female mice exhibited disrupted spatial reference memory. This phenomenon was somewhat more pronounced in *Abca7^−/−^* females. Female *Abca7^−/−^* mice also exhibited a moderate longer-lasting response to the locomotor-stimulating effects of MK-801 treatment than control females.

Our study expands on the initial finding that *Abca7* deficiency does not result in a remarkable physiological phenotype (i.e. unaltered food consumption, body weight development and weight of major organs; [5]), as our baseline tests revealed normal neurophysiological functioning of *Abca7* null mice and no gross behavioural abnormalities in locomotion, exploration and anxiety.

We investigated the cognitive phenotype of *Abca7* null mice as human research has linked common variants in *ABCA7* to AD [10]. The test battery incorporated tests for short-term memory of context familiarity (Y-maze), object recognition, amygdalar and hippocampal associative learning and spatial reference memory. *Abca7* ^−/−^ mice exhibited a task- and moderate sex-specific impairment of cognitive abilities. Male *Abca7* null mice showed disrupted object recognition (a better performance of females in a visual memory task has also been shown in other studies and has been related to sex hormones [16], [17]). Female knockout mice on the other side exhibited impaired spatial reference memory. It has been found that male rats perform better in reference memory, when it has been differentiated from the working memory aspects of a paradigm [18] and mouse studies have discussed reduced reference memory of females in relation to eostradiol levels [19], [20]. The cognitive impairments of *Abca7* deficient mice are in line with the expression profile of ABCA7, which is abundant in hippocampal and cortical neurons [5], [6], suggesting a potential moderate role of the ABC transporter in cognitive domains. Importantly, the impact of *Abca7* on cognition cannot be generalised across different ABC transporters: for example, the Abcg1 transporter is not involved in cognitive functioning as mice selectively overexpressing *Abcg1* performed equivalently to their WT littermates in the spatial reference and working memory version of the Morris water maze [21], which is a paradigm similar to the cheeseboard test [22].

Interestingly, validated mouse models for familial AD have shown disrupted spatial reference memory and object recognition [23]–[25] and deficiency in *Abca1*, which is one of the main ABC transporters and essential for cholesterol homeostasis, exacerbated amyloidogenesis in amyloid mouse models of AD [26], [27], whereas *Abca1* overexpression ameliorated amyloid load [28]. Another study reported that agonists of liver X receptors reduce Aβ levels and improve cognition in AD mice but only in the presence of ABCA1 [29]. As ABCA7 has the highest homology of all ABC transporters to ABCA1, it appears logical to investigate *Abca7*’s role in amyloid pathology as well. Indeed, our earlier *in vitro* work found that ABCA7 stimulates cholesterol efflux to apoE discs, regulates processing of amyloid precursor protein and inhibits the generation of neurotoxic Aβ peptides [11]. Future research should focus on the role that *Abca7* may play in cognition in established mouse models for AD.

A SNP of *ABCA7* has been found to be associated with schizophrenia [7] and ABCA7’s role in phagocytosis might impact on the deregulation of apoptosis in cortical regions of schizophrenia patients [8], [9]. The defects in the apoptotic pathway of schizophrenia patients may be a factor contributing to dysfunctional autoimmune system and elevated inflammatory cytokines observed in these patients [7]. Thus, we tested *Abca7* null mice in behavioural tasks relevant to schizophrenia: *Abca7* deficient mice showed unaltered baseline locomotion and sensorimotor gating but female *Abca7* ^−/−^ appeared to have longer-lasting susceptibility to the locomotion-stimulating effects of acute MK-801 treatment, which might indicate changes to the glutamatergic system, the processing of MK-801, or more specifically the NMDA receptor sensitivity/expression levels. Sexually dimorphic effects of MK-801 have been described by colleagues in the past [30]. Importantly, changes to the glutamatergic system are in line with the hypoglutamatergic hypothesis of schizophrenia [31].

The moderate behavioural differences detected between male and female *Abca7* ^−/−^ mice are consistent with sex-dependent effects of ABCA7 on mouse physiology (i.e. less visceral fat, lower serum and high-density lipoprotein cholesterol levels in female but not male knockouts [5]).

At this present stage the exact function of *ABCA7* is not fully defined. The initial identification of ABCA7 in hemopoietic cells pointed towards a role for regulating macrophage lipid efflux in particular [32]. Furthermore, given the homology of ABCA7 with ABCA1 it was predicted that ABCA7 would mediate cholesterol efflux. Most studies have confirmed that ABCA7 mediates phospholipid efflux, however ABCA7 is not as effective at mediating cholesterol efflux [33], [34]. In contrast to this expectation, macrophages isolated from *Abca7* ^−/−^ mice exhibited normal cholesterol and phospholipid efflux thereby indicating that ABCA7 may have another distinct biological function [5]. More recent work identified a role for ABCA7 in host defence system [35], [36]. The available evidence suggests that macrophage ABCA7 plays a key role in phagocytosis of apoptotic debris [36]. Because microglia actively phagocytose apoptotic debris in the brain [37], and data from our own laboratory indicates that ABCA7 has the highest expression in human microglia [6], a function for microglial ABCA7 in phagocytosis of apoptotic debris is also plausible. Unrelated to the lipid efflux activity, ABCA7 may modulate APP processing by altering the trafficking of APP with low-density lipoprotein receptor-related protein to the cell surface [36]. Furthermore, two splice variants of *ABCA7* detected in the human brain in one study may have different biological functions [38]. The so-called type II ABCA7 isoform appears to be localised in the endoplasmic reticulum, unlike the major isoform, which is predominantly localised to the plasma membrane.

Based on the high level of homology between ABCA7 and ABCA1, published data on behavioural effects of *Abca1* deficiency may shed more light on the impact of ABC transporters on behavioural domains and the potential mechanisms. Cholesterol homeostasis changes in Cre-lox *Abca1* mutant mice were mirrored by disturbances to motor activity and improved sensorimotor gating [39]. However, Cre-lox mice showed normal cognitive abilities in the Morris water maze. Thus, the behavioural phenotype of *Abca7* ^−/−^ mice appears quite different to what has been described for *Abca1* ^−/−^ mice. Importantly, *in vitro* work confirms that ABCA7 does not recapitulate ABCA1 function but rather has distinct activities on apolipoprotein-derived generation of high-density lipoprotein [40] and cellular phagocytotic function [9]. Furthermore, *Abca7* null mice have no defect in apolipoprotein-stimulated sterol or phosphatidylcholine transport, suggesting that cholesterol and phosphatidylcholine are not likely to be the primary physiological substrates of ABCA7 transporter activity [5].

In conclusion, this study is the first to provide a detailed analysis of *Abca7* function in behavioural domains relevant to neurodegenerative diseases *in vivo*. *Abca7* appears to impact on particular aspects of cognitive functioning and to have a subtle effect on the locomotor response to NMDA blockage. Further research will clarify, if therapeutic strategies targeting ABCA7 might be beneficial in improving cognitive functioning in AD.

## Materials and Methods

### Animals

The generation of *Abca7* null (*Abca7* ^−/−^) mice, which were generously provided by Prof. Mason W. Freeman (Centre for Computational and Integrative Biology, Massachusetts General Hospital, Harvard Medical School), has been described elsewhere [5]. Test mice were adult (males: 19±3 weeks; females: 20±4 weeks) cohorts of male and female *Abca7* ^−/−^ mice and wild type-like control littermates (WT), which had been backcrossed for 15 generations onto a C57BL6/J background (males: *Abca7* ^−/−^ = 11 and WT = 10; females: *Abca7* ^−/−^ = 8 and WT = 15). Mice were bred and housed in independently ventilated cages (Airlaw, Smithfield, Australia) at Animal BioResources (Moss Vale, Australia). Two weeks before testing mice were transported to Neuroscience Research Australia (NeuRA), where they were pair-housed in Polysulfone cages (1144B: Tecniplast, Rydalmere, Australia), which were enriched with certified polycarbonate mouse igloo (Bioserv, Frenchtown, USA), tissues for nesting material and a steel ring in the cage lid. Mice were kept pair-housed under a 12: 12 h light: dark schedule [light phase: white light (illumination: 124 l×) – dark phase: red light (illumination: <2 l×)]. Food and water were available *ad libitum*.

#### Ethics statement

Research and animal care procedures were approved by the University of New South Wales Animal Care and Ethics Committee in accordance with the Australian Code of Practice for the Care and Use of Animals for Scientific Purposes.

### Behavioural Phenotyping

Animals were tested in a battery of behavioural tasks relevant to schizophrenia and cognition, which are well-established at Neuroscience Research Australia (the least aversive/disruptive tasks were carried out first) [15], [41], [42]: elevated plus maze, Y maze, novel object recognition task, fear conditioning, prepulse inhibition, open field [baseline and after treatment with non-competitive N-methyl-D-aspartate (NMDA) antagonist MK-801] and cheeseboard using an inter-test interval of at least six days. All devices (and objects) were cleaned thoroughly with 70% ethanol in between trials and sessions. All testing occurred during the light phase (within 1–5 h of light onset) with the exemption of the elevated plus maze, which was run during the dark phase (2 h prior to light onset). An additional male cohort (N: *Abca7* ^−/−^ = 6 and WT = 6) was tested for motor functions and coordination as well as sensory abilities to avoid that impairments in those domains result in false positive or negative phenotyping outcomes [12], [43].

#### Elevated plus maze (EPM)

Mice were allowed to explore the apparatus freely for 5 min (as described previously [43]). Arm entries (when the mouse entered an arm with all four paws), distance travelled, time spent in arms as well as the frequency of *rearing*, and *head dipping* were scored for the different arms. Anxiety-related behaviour was measured by time spent on open arms (open time) and open arm entries as a percentage of the total measures (open entry ratio), and the frequency of *stretch attend postures*.

#### Y-maze (YM)

The Y-maze assessed short-term memory of context familiarity [44]. Arms were equipped with different internal visual cues. Bedding covered the apparatus floor and was changed in between sessions. The Y-maze test consisted of two trials with a 30 min inter-trial interval (ITI). The trial duration for training and test was 10 and 5 min respectively. During training, one arm was blocked off (novel arm). In the test trial, all arms were accessible, and mice were allowed to explore the apparatus freely. Time spent and distance travelled was recorded for each arm using Any-Maze™ video tracking software (Stoelting Co., Wood Dale, USA). The percentage of time spent in the novel arm (novel time) was calculated using [(novel time/total time) × 100]. The corresponding calculation was performed for distance travelled in the novel arm (novel distance).

#### Novel object recognition task (NORT)

The distinction between familiar and unfamiliar objects is an index of recognition memory, and its measurement is aided by the innate preference of rodents for novel over familiar objects [45]. The NORT was conducted over 3 days; two trials (10 min per trial) were conducted per day with a 1 h ITI (for details see [15], [41], [42]). On the test day (following two days of habituation to the arena and the test procedure), mice were exposed to two identical objects in trial 1 (sample trial), and then one familiar and one novel object in trial 2 (test trial). Objects and their location were counterbalanced across genotypes. The frequency and duration of *nosing* and *rearing* the familiar and novel objects were recorded offline by a trained experimenter blind to the sex and genotype of the test animals using Any-Maze™ tracking software. The percentage exploration time (time spent *nosing* + *rearing* objects) for the novel object (% novel exploration) was calculated using [(novel object exploration time/novel + familiar object exploration time) × 100] and served as an indicator for short-term object recognition memory.

#### Fear conditioning (FC)

Fear conditioning (FC) is a form of associative learning that occurs when a previously neutral stimulus (e.g. tone) elicits a fear response after it has been paired with an aversive stimulus [46]. On conditioning day, animals were placed in the test chamber (Model H10-11R-TC, Coulbourn Instruments, Whitehall, USA) for 120 s. A 80 dB conditioned stimulus (CS) was presented for 30 s with a co-terminating 0.4 mA 2 s foot shock (unconditioned stimulus; US) twice with an inter-pairing interval of 120 s. The test concluded 120 s later. On day 2 (context test), the animals were returned to the apparatus for 7 min. On day 3 (cue test), animals were placed in an altered context for 9 min. After 120 s (pre-CS/baseline), the CS was presented continuously for 5 min. The test concluded after another 120 s without the CS. Time spent *freezing* was measured using Any-Maze™ software [15], [41], [42].

#### Prepulse inhibition (PPI)

PPI is an operational measure of sensorimotor gating, which is impaired in schizophrenia patients [47], [48]. Startle reactivity was measured in millivolts using SR-LAB startle chambers (San Diego Instruments, San Diego, USA). The PPI test consisted of 5 min acclimatisation to 70 dB background noise, followed by 121 trials in a pseudorandom order: 5×70 dB trials (background); 5×90 dB trials; 15×120 dB trials (startle) and 96 trials comprising a prepulse of either 74, 82 or 86 dB presented 32, 64, 128, or 256 ms (variable interstimulus interval; ISI) prior to a startling pulse of 120 dB (PPI response) (for further details see [49]). The blocks of startle responses at the beginning, middle and end of the PPI protocol (averaged across 5 trials each) were used for ASR habituation. Percentage PPI (% PPI) was calculated as [(mean startle response – PPI response)/mean startle response] × 100. % PPI was averaged across ISIs to produce a mean % PPI for each prepulse intensity.

#### Open field (OF)

General motor activity was evaluated by placing the mouse into an infrared photobeam controlled open field activity chambers (41 cm × 41 cm; Tru-Scan Photo Beam Activity System: Coulbourn Instruments). Animals were tested for 30 min (baseline) before MK-801 (0.25 mg/kg body weight; dissolved in saline) was administered i.p. (injection volume: 10 ml/kg body weight). Following the injection, animals were put back into the OF chambers for another 60 min (MK treatment). Distance travelled and *rearing* were measured using Any-Maze™ software [15], [41], [42].

#### Cheeseboard (CB)

The cheeseboard (CB) paradigm was employed as a dry-land equivalent of the Morris water maze [15], [22], [50]. Mice were trained to find a food reward over a number of days; reference memory was indexed by a decreased latency to find the reward over days. During habituation (three days to the blank side of the CB) two 2 min trials were conducted each day with a 10 min ITI. Mice were food-restricted (starting 24 h before the first habituation session) and kept at 85–90% of their pre-test body weight throughout testing (mice were fed for 1–2 h per day). *Task Acquisition/Spatial Reference Memory Training:* Mice were trained over 15 days (three trials per day with a 10 min ITI) to locate the food reward. The location of the target well (counterbalanced across genotypes) was kept constant for each mouse between trials and across days. If the target well was not located within 2 min, mice were placed next to the target well and allowed to consume the food reward. *Spatial Reference Memory:* A probe trial was conducted on day 9 (males and females) and day 16 (males only), where no wells were baited and mice were given 2 min to explore the board freely (probe trial 16 results are shown for males as no clear target preference was detected on day 9). For this, the board was divided into 8 zones corresponding to each line of 4 caps as well as a centre zone (40 cm diameter = start zone). The time spent in each zone was measured using Any-Maze™. Data are presented for ‘% target zone time’, which calculates the time spent in the target zone as a percentage of time spent in all zones (minus the time spent in the start zone).

### Statistical Analysis

Results were analysed using two-way analysis of variance (ANOVA: between factor: ‘genotype’ and ‘sex’) followed by one-way ANOVA split by corresponding factor(s) where appropriate as published previously [51], [52]. Three-way repeated measures (RM) ANOVAs were used to control for successful learning (Y-maze: ‘arm type’, NORT: ‘object’), for effects across trial types (FC: ‘first 2 min’, CB: ‘day’, PPI: ‘ASR block’ and ‘prepulse’), or for effects over time (OF: ‘5 min block’, FC: ‘1 min block’), which were followed by simple contrasts where appropriate. For the cheeseboard probe trials, single sample t-tests were used to assess if % target zone time was greater than chance (i.e. 12.5%). The physical exam data were analysed using one-way ANOVAs (between factor: ‘genotype’). Analyses were conducted using SPSS for Windows 19.0. Differences were regarded as significant if *p*<.05. In line with Rothman and Perneger the data were not adjusted for multiple comparisons and were interpreted as such in the discussion [53], [54]. All data are presented as means ± standard error of the mean (SEM). Significant one-way ANOVA effects of *Abca7* ^−/−^ *versus* WT of the corresponding sex are indicated by ‘*’ (*p<.05, **p<.01 and ***p<.001) and significant effects of females *versus* males of the corresponding genotype are indicated by ‘#’ (^#^ *p*<.05, ^##^ *p*<.01 and ^###^ *p*<.001).
